# Biosensor-Integrated Drug Delivery Systems as New Materials for Biomedical Applications

**DOI:** 10.3390/biom12091198

**Published:** 2022-08-29

**Authors:** Iwona Cicha, Ronny Priefer, Patrícia Severino, Eliana B. Souto, Sona Jain

**Affiliations:** 1 Cardiovascular Nanomedicine Unit, Section of Experimental Oncology and Nanomedicine, University Hospital, Friedrich-Alexander-Universität Erlangen-Nürnberg, 91058 Erlangen, Germany; 2 Massachusetts College of Pharmacy and Health Sciences, Boston University, Boston, MA 02115, USA; 3 Post-Graduation Program in Industrial Biotechnology, University of Tiradentes, Aracaju 49010-390, Sergipe, Brazil; 4 Institute of Technology and Research, University of Tiradentes, Aracaju 49010-390, Sergipe, Brazil; 5 Department of Pharmaceutical Technology, Faculty of Pharmacy, University of Porto, 4200-135 Porto, Portugal; 6 REQUIMTE/UCIBIO, Faculty of Pharmacy, University of Porto, 4200-135 Porto, Portugal

**Keywords:** biosensors, drug delivery systems, smart polymers, biomedical applications, chronic treatments

## Abstract

Biosensor-integrated drug delivery systems are innovative devices in the health area, enabling continuous monitoring and drug administration. The use of smart polymer, bioMEMS, and electrochemical sensors have been extensively studied for these systems, especially for chronic diseases such as diabetes mellitus, cancer and cardiovascular diseases as well as advances in regenerative medicine. Basically, the technology involves sensors designed for the continuous analysis of biological molecules followed by drug release in response to specific signals. The advantages include high sensitivity and fast drug release. In this work, the main advances of biosensor-integrated drug delivery systems as new biomedical materials to improve the patients’ quality of life with chronic diseases are discussed.

## 1. Introduction

Biosensor-integrated drug delivery systems have been extensively studied, especially for the treatment of chronic diseases such as cardiovascular diseases (CVD), diabetes mellitus, and cancer, where regular drug administration and continuous monitoring are relevant [1,2,3]. The conventional modes of treatment have been associated with serious side effects; thus, over the years, controlled drug delivery systems have been explored as a promising alternative to improve the efficacy and safety by optimizing the duration and kinetics of release [4]. Moreover, the use of systems that can initially sense markers associated with regenerative medicine and diseases, and subsequently release their payloads, has shown a great impact on the treatment of chronic diseases [5,6].

Biosensors are analytical devices composed of two main components: a bio-recognition element and a transducer [7]. The bio-recognition element of the sensor identifies the target analyte, while a transducer converts the result of the molecular recognition into an electrical signal. Different biomolecules such as enzymes, nucleic acids, antibodies, proteins, and peptides can be used as a bio-recognition element and biosensors can thus be used to detect specific physiochemical changes in the body (associated with the diseases) with high sensitivity and specificity [8]. Biosensors have been widely utilized for diagnostic and imaging [9,10,11], however, they are not originally equipped with therapeutics to treat the diseases. Several studies that merge biosensing and drug delivery concepts have been described in the last few decades [12,13,14]. These systems are a special class of biosensor designed for the continuous analysis of biological molecules followed by drug release in response to specific signals. These delivery systems, also known as closed loop delivery systems, have proven to be practical tools by tuning drug release as a function of specific signals associated with physiological and pathological processes [4].

The closed-loop drug delivery systems usually consist of a monitoring component that senses the surrounding conditions and an actuator component with the capability to trigger drug release. The pairing of the monitor/actuator architecture allows the drug release to be activated at or above a certain signal concentration or threshold, but inhibits such release when the signal level is in normal ranges [15]. A typical example of such systems is the glucose-responsive insulin delivery system, which imitates the pancreatic beta cells to release insulin with a specific dose at a specific time point by responding to the plasma glucose levels [16].

Many biosensor-integrated drug delivery applications utilizing bio microelectromechanical systems (bioMEMS), electrochemical sensors, and stimulus responsive biopolymers have been described. MEMS are devices with electrical and mechanical components. MEMS designed for biomedical applications are called bioMEMS, which have gained much attention in the biomedical engineering field for biomolecular analyses and sensing. BioMEMS provide many advantages such as short response time, high scalability, and high sensitivity. In bioMEMS, physical, chemical, or biological signals are converted into electrical signals that trigger the drug release. BioMEMS are implanted into the human body and the drug is released according to sensor feedback [17,18].

Electrochemical biosensors have electrodes that convert the chemical signal into an electrical signal. Electrochemical sensors can detect various biomolecules in the human body such as glucose, cholesterol, uric acid, lactate, DNA, hemoglobin, blood ketones and have great potential to treat diseases related to imbalances of biomolecules. Electrochemical sensors are mostly used for biosensing applications, with very few studies relating to biosensing integrated drug delivery applications [9,19].

Bioresponsive polymers or smart polymers can undergo structural alterations in response to physical, chemical, or biological stimuli. Many microdevices making use of these smart polymers have been described, which respond to external stimuli and deliver drugs when required [20,21]. These smart polymer-based systems, although not true biosensors (as they lack the signal processing unit), have been widely studied for biosensing integrated drug delivery systems. Attachment of the enzyme glucose oxidase and insulin within a hydrogel, which is responsive to pH changes, is a particularly good example of one such system where the smart polymer acts both as a sensor of glucose concentration and as a drug delivery vehicle for insulin [22].

In this review, we have brought together the exciting advances in the field of biosensor integrated closed-loop drug delivery systems. We discuss the bioresponsive smart polymers, bioMEMS, and electrochemical sensors described in the literature, focusing on diabetes, cancer, cardiovascular diseases, and regenerative medicine.

## 2. Applications in Diabetes

One of the most recognized diseases worldwide is diabetes. There are currently almost half a billion individuals globally with this disease and this is expected to crest three quarters of a billion by the end of the decade [23]. Traditionally, diabetes is broken into three categories: Type 1 (previously referred to as juvenile); Type 2 (occasion defined as adult onset), and gestational diabetes [24]. Gestational diabetes occurs in ~2–10% of pregnant women with roughly 50% of these cases leading to the mother developing T2D after giving birth [25]. Generally, 5–10% of cases of diabetes are of the T1D form with the remaining 90–95% having T2D [25]. The need to monitor blood glucose, whether for T1D or T2D, is vital for the health and welfare of those afflicted with these diseases. Equally, if not more important, is the need to administer the necessary drug once the knowledge of one’s blood glucose is determined. It is this key second part that has led to significant efforts and ultimate successes in bringing closed-loop systems for diabetes management to market.

### 2.1. Urine-Glucose Testing

**Figure 1:**
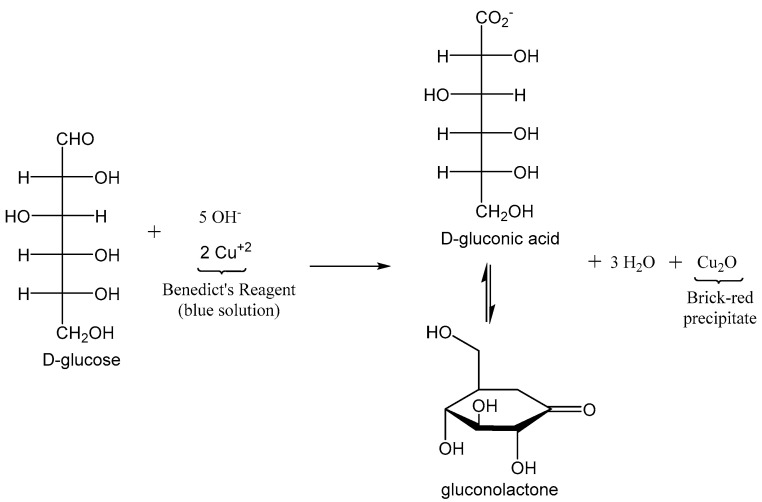
Benedict’s reaction for urine glucose testing.

Between the 1920s and 1960s, a patient’s urine was the only means by which to gauge one’s blood glucose. This required taking a few drops of urine and mixing in with Benedict’s solution to yield a bright red precipitate as an indicator that there was glucose in the sample (Figure 1) [26].

Beyond the lack of practicality of this approach, which was improved on with the “dipstix” [27], it was still only a proxy for blood glucose levels. Currently, there are a number of urine-glucose tests available commercially. However, the technology has its limitations, primarily, it is still simply a proxy for blood glucose. A recent report highlighted this issue, whereby the urine-glucose test was only 14% selective and even failed to identify ~16% of participants with diabetes [28]. A benefit that should not be overlooked with urine-glucose testing is the lack of potential infections that have been reported, albeit minimally, with blood glucose [29]. In developing countries, where blood transmitted pathogenic disease is more prevalent, the cost associated with the lancets themselves are a significant hurdle.

### 2.2. Blood Glucose Testing

In 1964, Dextrostix, by the Ames-Miles Laboratories, was developed as the first blood glucose test strip [30]. Similar to its predecessor, this approach utilized a colorimetric change, albeit enzymatically. Taken from Clinistix, which was developed in the 1940s, this double sequential enzymatic reaction proceeded by the initial conversion of glucose to gluconic acid (which is in equilibrium with gluconolactone) by glucose oxidase, which also yielded hydrogen peroxide [30]. The hydrogen peroxide acted as a reagent in the oxidation of *o*-toluidine, which was facilitated by peroxidase. The major advance with Dextrostix was the ability to trap the red blood cells by a semipermeable membrane to prevent interference. For its time, it was a revolutionary technology. However, by today’s standards, it would be considered somewhat archaic. In addition to requiring 1 min and a relatively large blood sample (30 μL), the results were gauged by the patient’s interpretation of a colorimetric change [30]. Fortunately, over the past decades, significant advances within nanotechnology have allowed for the self-monitoring of blood glucose to become a more manageable, less invasive, and expeditious process.

**Figure 2:**
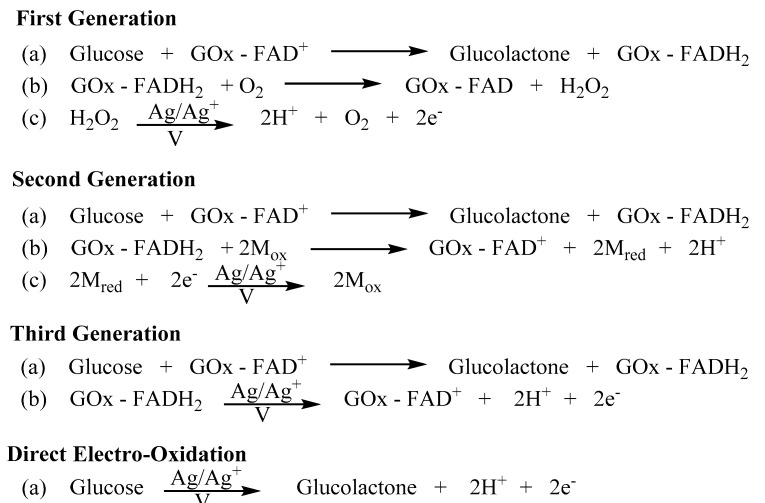
The steps involved in all generations of glucose monitoring via amperometric biosensors.

The most instrumental advancement with regard to blood glucose monitoring was the nanotechnology approach for both the creation of enzyme-based circuitry and the miniaturization of the necessary electrodes for the detection of an electrochemical oxidation/reduction potential. Unlike Dextrostix, the majority of today’s blood glucose detection devices employ a single enzymatic reaction. With just a single drop of blood, the glucose held within is reacted with a nanolayer of glucose oxidase that is complexed with its redox cofactor, flavin adenine dinucleotide (FAD) [31,32]. In this process, the glucose is oxidized to gluconolactone while the glucose oxidase-flavin adenine dinucleotide (GO_x_-FAD^+^) is reduced to GO_x_-FADH_2_. Upon the regeneration of GO_x_-FAD^+^ by the reaction with O_2_, also held within the blood, hydrogen peroxide (H_2_O_2_) is produced. The aforementioned nanolayer of GO_x_-FAD^+^ is coated on a silver working electrode surface. Thus, when the generated H_2_O_2_ is oxidized to 2H^+^ and O_2_, the corresponding amperometric signal can be correlated with the initial glucose concentration [31,32]. Although this first-generation electrochemical detection technology still dominates the blood glucose monitoring industry, three new generation of devices have been developed (Figure 2) [30].

The second- and third-generation of the electrochemical detection of blood glucose both employ the same nanolayer of GO_x_-FAD^+^ for the conversion of glucose to gluconolactone with the production of GO_x_-FADH_2_. For second-generation devices, instead of employing the natural co-substrate of molecular oxygen for the production of H_2_O_2_, these employ an impregnated artificial metal mediator [33,34,35,36]. Thus, in the oxidation of GO_x_-FADH_2_ back to GO_x_-FAD^+,^ the metal mediator is reduced. It is this reduced metal that when oxidized back by the silver working electrode yields a quantifiable amperometric signal [33,34,35,36]. With third-generation electrochemical glucose detectors, the metal mediator has been completely removed. In this case, the nanolayer has been covalently or electrochemically bound to the silver working electrode [37,38]. Thus, the conversion of GO_x_-FADH_2_ back to GO_x_-FAD^+^ can be accomplished by direct electron transfer. Unlike the prior two generation devices, this approach measures reduction as opposed to the oxidation potential. Various other enzymatic approaches have also been evaluated that have employed either glucose [39,40,41] or quinoprotein-based glucose dehydrogenases [42,43,44] in the aforementioned generations. Typically, these utilize mediators such as nicotinamide adenine dinucleotide (NAD^+^) and quinones.

The final electrochemical glucose detection technology, known as direct electro-oxidation (rarely referred to as fourth generation) is a non-enzymatic approach. Nanostructured electrodes, whether platinum–lead alloy nanowires [45], gold nanoparticles [46], platinum nanoforests [47], or general alloy nanostructures [48] such as those containing gold, palladium, rhodium, lead, or platinum work by simply measuring the electro-oxidation of glucose to gluconolactone. The advantage of this approach is that the incredible high surface area of these nanostructures allows for the remarkable electro-catalytic activity.

These fantastic advancements in the realm of nanotechnology have been translated from the aforementioned self-monitoring of blood glucose (SMBC) [49] to the newer continuous glucose monitors (CGMs) [50]. It was the development and adoption of CGMs, the first of which was by Metronic in 1999, that led to the closed-loop system finally being introduced into the market. However, it took almost two decades before an all-in-one device was actually approved. A primary concern with CGMs has been the “lag-time” related to the fact that these devices detected glucose changes in interstitial fluid as opposed to directly within the blood. This lag-time could be anywhere between 4 and 27 min. Regardless, it is the nano-sensing of CGMs coupled with traditional administration, whether insulin or glucagon, via an incorporated syringe-like injector system that has brought about these novel closed-loop delivery systems [29]. These devices are of great assistance in the health and wellness of the diabetic community. However, whether SMBC, CGM, or closed-loop systems all still have the major limitation of being invasive in nature and, although rare, a potential site of infection [29]. Thus, significant work has been underway to develop non-invasive approaches to monitor glucose levels, with many utilizing some nanotechnology in the hopes of also linking developing less-invasive closed-loop delivery systems.

### 2.3. Non-Invasive Glucose Testing

Although by definition urine glucose testing is non-invasive, it has been socially deemed untidy and is a relatively unpopular approach for diabetes management [51]. Currently, numerous novel, albeit not yet marketed approaches have been attempted that are looked at through the skin, tears, and even the breath for the monitoring of blood glucose. For through-skin approaches, near-infrared [52,53], mid-infrared [54,55], thermal emission spectroscopy [56,57], ultrasound [58,59], metabolic heat conformation [60,61], electromagnetic [62,63], mm-wave radar [64,65], and microwave sensing [66] have all been attempted with various degrees of success. More recently, tears have become an active area of research due to the fact that the leakage of glucose directly into tear fluid is known to occur either from the interstitial fluid or epithelial cells [67]. Thus, attempts utilizing the aforementioned nanotechnology enzymatic detection approaches have been evaluated in addition to some unique methods such as optical polarimetry [67,68] and retina pigmentation regeneration [69].

For breath detection, glucose is not the chemical that is being quantified. Instead, due to the propensity of individuals that have diabetes to undergo ketoacidosis, there is an inherent elevated level of ketones within the body, specifically acetone. Thus, researchers have utilized traditional technologies such as gas-chromatography, mass spectrometry, and/or combinations thereof to detect this volatile organic compound (VOC). Chitosan-based sensors have been developed for the detection of low concentrations of acetone, down to 0.1 ppm [70]. Unfortunately, the impediments of this technology are its (1) variability with humidity; (2) lengthy processing steps of making the film sensor, which included hydrofluoric acid, surface oxidation at temperatures > 1000 °C, photolithography, etc.; and (3) lack of selectivity to only acetone, as it also detected another volatile breath organic, methanol. Others have utilized Si-doped WO_3_ nanoparticle films [71]. This approach gives accurate and precise results, but variability in relative humidity, need to analyze at 400 °C as well as some response to ethanol are all limitations that need to be overcome. Similar successes with thin-walled SnO_2_ functionalized with Pt nanoparticles have been reported [72], however, the high temperature requirements as well as the lack of selectivity (also detects toluene) have hindered its further development. Others have examined Pt-functionalized WO_3_ nano-hemitubes that require 300 °C and lack selectivity (H_2_S and toluene are also detected, albeit not to the intensity of acetone) [73]. Fe_2_O_3_ doped with Pt as semiconductors has shown success, however, a >200 °C temperature was needed, leading to a decrease in its long-term stability [74]. More recently, cavity enhanced absorption spectroscopy (CEAS) has gained significant attention [75]. However, this type of device tends to be 1–2 feet in length, requires a vacuum pump, and is currently not economical. Applied Nanodetectors is attempting to launch a product to detect the breath acetone levels using a headspace VOC analysis [76].

Recently, a unique approach employed the layer-by-layer self-assembly of multilayers utilizing the pseudo-polyelectrolyte, poly (4-vinylbenzeneboronic acid) (PVBBA) with the weak polyelectrolyte, poly (allylamine hydrochloride) (PAH) to create nanofilms for the detection of breath acetone [76]. The PVBBA/PAH nanofilms are coated on UV-transmitting poly(methyl methacrylate) (UVT-PMMA) at different assembly pH and layer numbers. The slides are subjected to a light emitting diode with a peak wavelength of ~300 nm and detected via a UV-photosensor with an integrated transimpedance amplifier. Upon the exposure of a 10-layered PAH/PVBBA coated UVT-PMMA slide to acetone vapor, crosslinking occurred via a Petasis reaction. These results suggest that it may be possible to quantify this reaction and therefore obtain accurate acetone concentrations. Indeed, clinical studies have shown that this nanotechnology may be a viable approach to bring the first non-invasive diabetes monitor to market [77].

Just as SMBC led to CGMs, which has allowed for the delivery of the vital insulin drug directly into the body on-demand [50], it is the desire within the non-invasive biotechnology industry to duplicate this pathway. A future where one could simply breathe, blink, or sweat to obtain a reading of one’s glucose levels that when sent to a receiver to administer the correct type and amount of drug is a future worth working toward.

## 3. Applications in Cancer Treatment

Tumor physiology differs markedly from normal tissue physiology. The microenvironment of tumor tissues is associated with areas of O_2_ depletion, mild acidity, high GSH concentration, and elevated level of hyaluronidase, which can serve as endogenous stimuli. Many bio-responsive drug delivery systems involving pH-, redox-, enzyme-response, and the expression of tumor associated markers (e.g., miRNA, nucleolin) have been described, which show a high potential for cancer treatment [77]. For example, tumor cells show an exaggerated demand for glucose to meet their energy demand for growth compared to normal cells. Glucose oxidase (GOx, which specifically catalyzes beta-d-glucose oxidation into gluconic acid and hydrogen peroxide (H_2_O_2_) by using molecular oxygen as an electron acceptor) has thus been strategically studied for noninvasive cancer diagnosis and therapeutics by biosensing glucose levels by measuring the oxygen consumption, pH changes, or production of H_2_O_2_ associated with drug release. GOx has also been combined with other enzymes, hypoxia-activated prodrugs, photosensitizers, or Fenton’s reagents to generate multi-modal synergistic cancer therapies [78]. The biosensing integrated drug delivery systems reported for cancer will be described in this section.

### 3.1. Stimuli-Responsive Polymers

Stimuli-responsive polymers act as an essential constituent of nanoscale sensor-like delivery platforms and have been most extensively explored in the case of cancer. Various chemical (different redox potential of redox couples such as glutathione/glutathione disulfide), physical (temperature and pH), and biological cues (e.g., enzymes, adenosine-5′-triphosphate (ATP), and nucleic acids) specific for the tumor microenvironment (TME) have been used to stimulate these polymers to deliver their payload, which will be the focus of this section.

#### 3.1.1. Gox-Based Systems

**Figure 3:**
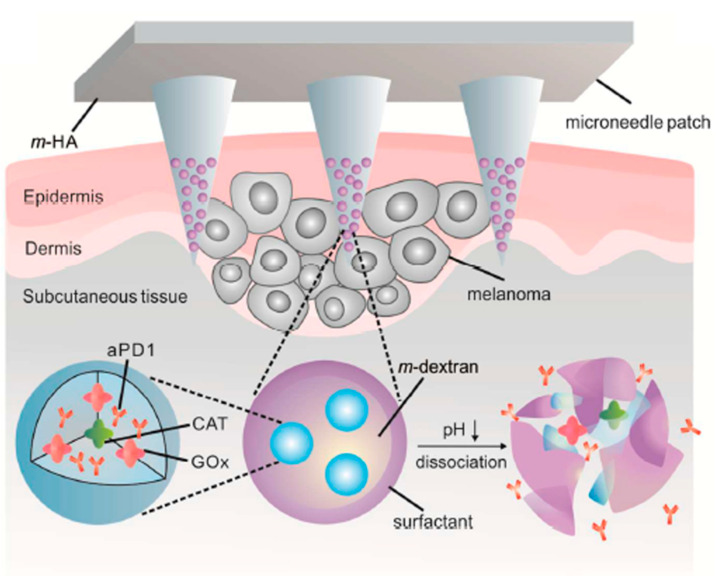
A schematic representation of anti-PD-1 (programmed death-1 pathway) (aPD1) delivery via microneedle patch loaded with nanoparticles. The glucose oxidase/catalase (GOx/CAT) enzymatic system was immobilized inside the nanoparticles, which resulted in the conversion of blood glucose to gluconic acid, resulting in the dissociation of nanoparticles, subsequently leading to the release of aPD1. Reproduced from Wang et al. [79], Open Access.

The presence of gluconic acid because of GOx-catalyzed glucose oxidation increases the acidity of TME; this increase can be used to construct pH-sensitive and glucose-sensitive drug delivery systems. Wang et al. [79] described a self-degradable microneedle patch for the delivery of anti-PD-1 (programmed death-1 pathway). The microneedle was composed of biocompatible hyaluronic acid (HA) integrated with pH-sensitive dextran nanoparticles that encapsulated the anti-PD-1 and GOx-CAT (catalase) enzyme system. GOx in the microneedle converted glucose to gluconic acid to generate an acidic environment, which promoted the self-dissociation of nanoparticles and the release of anti-PD-1 (Figure 3). CAT assisted glucose oxidation by the regeneration of O_2_ and helped consume undesired hydrogen peroxide (H_2_O_2_). The patch described by the authors could painlessly penetrate the epidermis and become submerged in the interstitial fluid to efficiently deliver its payload to the tumor microenvironment. In vivo studies using mouse models with melanoma showed that a single administration of the microneedle patch inhibited tumor growth superior to those obtained with intratumor injection of the same dose. Su et al. [80] described glucose oxidase (GOx) triggered gelation of *N*-hydroxyimide–heparin conjugates to form enzyme-responsive hydrogels for cell-specific drug delivery. *N*-hydroxy-5-norbornene-2,3-dicarboximide (HONB) can be reduced by D-glucose in the presence of GOx, forming a carbon-centered radical. In this work, the heparin-HNOB conjugate anchored to doxorubicin (DOX) was subjected to GOx mediated polymerization to form a hydrogel at room temperature. Heparin is a highly sulfated polysaccharide belonging to the glycosaminoglycan (GAG) family. Depending on the concentration of heparinase (associated with tumor angiogenesis and metastasis), the DOX bound heparin chain encapsulated in the hydrogel could be cleaved. Close to 46% of the drug release within 60 h was confirmed in the presence of 5 U/mL of heparinase, whilst no release was observed from the control gel. Cell toxicity was analyzed in three cell lines (HeLa, HepG2, and NIH-3T3) and presented positive results in all three cases. Hep (DOX)SN (drug-loaded gel) was able to release the drug in a cell-specific manner by responding to the environmental levels of heparinase. The platform described by the authors could be used to target cancer cells overexpressing heparinase. The use of this gel also minimized the adverse effects of premature drug release on normal cells. These enzyme responsive hydrogels have the potential to serve as smart, multifunctional platforms for targeted cargo and regenerative medicine.

#### 3.1.2. Redox Responsive Systems

**Figure 4:**
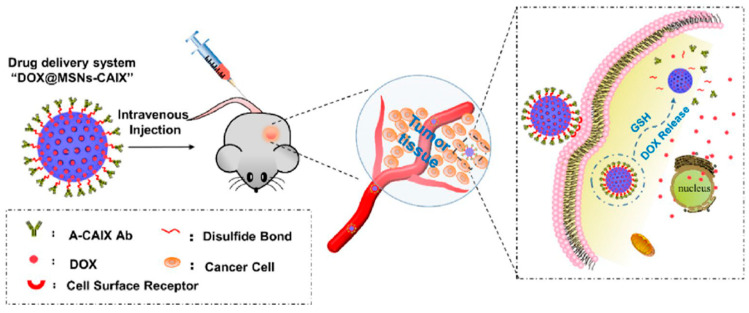
The A-CAIX Ab targeted mesoporous silica nanoparticles utilized as a redox-responsive drug delivery system. Reproduced from Chen et al. [81], Open Access.

The presence of GSH in tumor cells has been reported to be four-fold higher than that of normal cells. Chen et al. [81], taking advantage of this tumor microenvironment, developed a redox-responsive drug delivery system where mesoporous silica nanoparticles (MSNs) were used as the vehicle to load DOX. Furthermore, anti CAIX antibody (CAIX is highly expressed in various cancer tumors) was surface functionalized on mesoporous silica nanoparticles via redox-responsive disulfide linkages (Figure 4). The presence of GSH contributed to the cleavage of disulfide bonds between the anti CAIX antibody (A-CAIX Ab) and MSNs, which resulted in the release of DOX loaded on MSNs. The authors reported that the release was pH-dependent, where a lower pH resulted in a higher release amount. The pH value of extracellular tumor tissues (6.5–6.8) tends to be more acidic than that of the normal tissues (7.4) and further decreases to 4.5–5 in the lysosomes and 5.5–6.0 in endosomes. Thus, an increased release in an acidic environment favors treatment targeting the tumor. The authors reported that, in vivo, the DOX@ MSNs-CAIX could suppress the tumor growth more efficiently. Compared with the CAIX negative mouse embryo fibroblast (MEF) cells, more DOX@MSNs-CAIX was internalized into the CAIX positive 4T1 cells (mouse breast cancer cells) and induced more tumor cell apoptosis.

#### 3.1.3. DNA-Based Systems

DNA molecules have proved to be excellent substrates for the design and development of molecular devices that sense and respond to external signals. These DNA-based robotics have been used as imaging probes as well as cargo delivery vehicles [82,83,84,85]. Li et al. [86] described a DNA nanorobotic system using DNA origami for the targeted delivery of thrombin to the tumors. The nanorobot was functionalized on the outside with DNA aptamers (AS1411, [87]) that bind the tumor specific protein nucleolin. Blood coagulation protease (thrombin) was attached to the inner surface of the tubular nanorobot. The binding of the aptamer to nucleolin opened the tubular nanocarrier, exposing the thrombin, which resulted in vascular occlusion and thus cell death at the tumor site. The authors confirmed the specific opening of the thrombin-loaded nanorobot in response to the target protein, and thrombin induced blood coagulation was confirmed in the in vitro experiments. Furthermore, using tumor-bearing mouse models, the authors showed that intravenously injected DNA nanorobots delivered thrombin specifically to the tumor associated blood vessels and stimulated intravascular thrombosis, resulting in tumor necrosis and the inhibition of tumor growth. The nanorobot also proved to be safe and immunologically inert in mice and Bama miniature pigs. The DNA nanorobot described in this study thus recognized tumor microenvironmental signals, which triggered nanostructural changes resulting in drug release. In the melanoma mouse model, the nanorobot not only affected the primary tumor, but also prevented the formation of metastasis, showing promising therapeutic potential. Such DNA nanorobotic systems, which prompt drug release on the recognition of specific biomarkers, can inspire novel cancer therapeutics associated with different targeting molecules to mediate the delivery of multiple biologically active compounds. Zang et al. [88] described the use of mesoporous silica nanocarriers loaded with DOX where the pores were capped with a DNA-based structure containing antisense oligonucleotides. The biosensing of oncogenic miRNA in the tumor tissues resulted in binding to the antisense oligonucleotide and the detachment of the DNA-based structure accompanied by DOX release.

#### 3.1.4. Other Polymer-Based Microdevices

**Figure 5:**
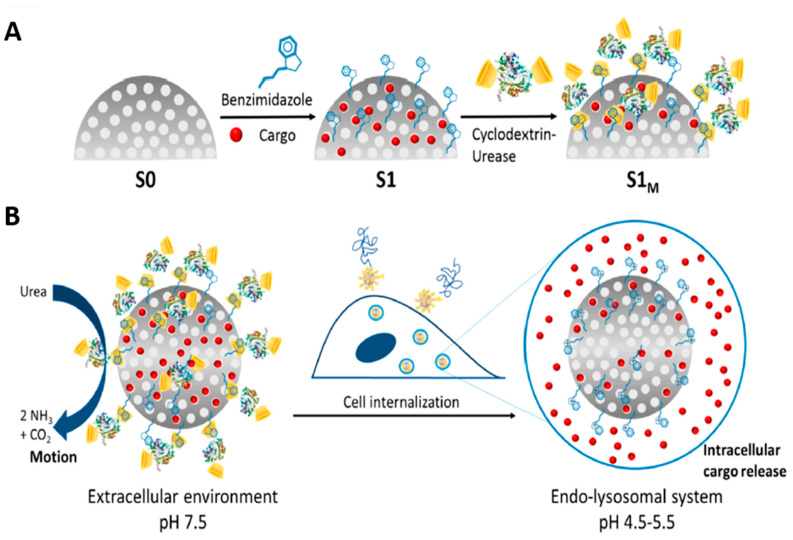
The development (**A**) and working (**B**) of the enzyme-powered stimuli-responsive nanomotors. Reproduced from Lopis-Lorente et al. [90], Open Access.

Dreaden et al. [89] described a tumor microenvironment-responsive layer-by-layer (LbL) polymer drug carrier targeting tumors via two independent mechanisms: the pH-dependent cellular uptake at hypoxic tumor pH and hyaluronan-directed targeting of the cell-surface CD44 receptor (highly expressed in tumor cells). Layer-by-layer (LbL) assembly is a simple method that makes it possible to design multilayer architectures with nanometer precision. The technique is based on the alternating adsorption of synthetic and natural polyelectrolytes and other components such as RNA and DNA, or nanoparticles on the surfaces. Two weak polyelectrolytes, hyaluronic acid (HA) and poly (l-lysine) (PLL), were used as functional biopolymers for the LbL nanoparticle architectures developed in this study. The PLL/HA-LbL nanoparticles undergo a pH-dependent structural transition at hypoxic tumor pH (i.e., 6.8 to 7.0) where swelling, loss of anionic charge, and decreased surface energy correlate with increased cell uptake. The authors reported targeted cellular delivery in vitro and in vivo, with effective tumor penetration and uptake. Furthermore, the nanoscale drug carriers selectively bound CD44 and diminished cancer cell migration in vitro while co-localizing with the CD44 receptor in vivo. In this work, multimodal targeting of LbL nanoparticles was thus shown to be a powerful strategy for tumor-specific cancer diagnostics and therapy, which can be accomplished using a single bilayer of polyamine and hyaluronan. Micro/nanomotors capable of self-propulsion (by converting the surrounding energies into mechanical motion) have been reported to cross biological barriers such as dense extracellular matrix, blood–brain barrier, and blood–tumor barrier in an autonomous manner boosting targeted delivery and deep tumor penetration. Lopis-Lorente et al. [90] designed enzymatic nanomotors based on mesoporous silica gated with pH-responsive nanovalves (Figure 5). The nanomotor described by the authors consist of mesoporous silica nanoparticles, functionalized with benzimidazole groups, loaded with drugs [Ru (bpy)_3_]Cl_2_, or dox, and capped with cyclodextrin-modified urease (CD-U), forming inclusion complexes between CD-U and the benzimidazole moieties. Urease resulted in increased Brownian motion of nanoparticles in the presence of urea (serving as biofuel). The benzimidazole: CD-U nanovalves served as caps that inhibited the cargo release at physiological pH. Cargo delivery took place only at acidic pH due to protonation of the benzimidazole groups and the subsequent dethreading of the benzimidazole: CD-U inclusion complexes. Using HeLa cells, the authors showed enhanced internalization in the presence of the urea. Furthermore, the payload was only released upon cellular uptake due to an acidic environment. In this study, the use of gated enzyme-powered nanomotors could improve the drug delivery properties. These findings may further motivate and inspire the development of micro- and nanomotors for the transport and stimuli responsive controlled release of drugs.

### 3.2. Implantable BioMEMS and Electrochemical Systems

Anti-cancer drugs often work on narrow effective therapeutic concentrations, above which they become toxic and below which are ineffective. By monitoring in vivo drug concentrations in real-time, a feedback system can continuously regulate the rate of drug delivery, making sure that the patient receives a dosage that minimizes the side-effects and toxicity while maximizing the therapeutic effectiveness. This kind of closed loop system is well-suited for diseases such as cancer, which require long sessions of intravenous delivery from automated infusion pumps, but to date, little work has been carried out in this area.

Mage et al. [91] recently presented an aptamer-based voltametric biosensor that enabled the first demonstration of the closed-loop control of doxorubicin in rabbits. This system consists of a biosensor, controller, and infusion pump, performs real-time processing of signals generated from an aptamer-based biosensor [92], and then uses the resulting data to achieve closed-loop feedback control of in vivo drug concentration. Briefly, this biosensor uses electrochemically tagged aptamer probes that are designed to undergo a reversible conformation change on binding to their drug target, causing a change in the redox current between the electrochemical tag and an electrode. This electrochemical measurement occurs within a microfluidic device that continuously samples a small volume of blood directly from the animal’s bloodstream, achieving rapid, quantitative, and specific measurement of the in vivo drug concentration. The authors demonstrated that the system could maintain the concentration of doxorubicin in live rabbits and rats at any desired set point and in real-time, while automatically compensating for pharmacokinetic differences among the individual animals. The sensor described by the authors was designed for ex vivo operation and requires catheterization of the patient for continuous blood flow, but the fundamental design is amenable to miniaturization and implantation with improvements in packaging. Moreover, these sensors could be adapted for the detection of any biomolecule for which an appropriate aptamer or enzyme isoform is available.

Implantable technology has also been described for the treatment of brain tumors [93,94,95]. The presence of the blood-brain barrier is a significant limitation to the development of more effective brain tumor therapies as it prevents the transfer of non-lipid soluble molecules and particles larger than 500 Da in size into the brain [96]. Systemic toxicity for commonly used chemotherapeutic agents is often reached before obtaining a therapeutically effective concentration in the brain. Masi et al. [97] developed a MEMS-based intracranial drug-delivery device to overcome the limitations of passive intracranial drug-delivery systems. The device proposed by the authors was developed to deliver the clinically utilized chemotherapeutic temozolomide (TMZ) in a rodent glioma model and consists of a liquid crystalline polymer reservoir, capped by a MEMS silicon microchip containing three nitride membranes that can be independently opened by applying a brief (0.4 ms) electrical pulse. This electrical pulse causes resistive heating, which causes expansion of the gold fuse and rupture of the underlying nitride membrane. The safety of implanting the device intracranially was confirmed with preliminary in vivo studies and the TMZ delivered from the device was effective at prolonging animal survival in a rodent glioma model.

Mounting evidence suggests that the tumor microenvironment contributes to drug response and resistance [85,86,87]. Short-term implantable devices have been developed to evaluate drug sensitivity within solid tumors. Jonas et al. [98] reported a microdevice (measuring 820 µm in diameter) containing multiple reservoirs (up to 16) that was capable of delivering drugs directly into the tumor regions at concentrations of one millionth of the systemic dose. The radiopaque device was delivered into tumors through a biopsy needle and allowed for the diagnostic assessment of each drug effect via standard ultrasound and computed tomography (CT) imaging. These types of devices with controlled drugs release directly within the TME, reduce potential toxic systemic side effects, and could be further explored coupled with the local tumor microenvironment and biomarkers. In addition, in highly toxic therapies such as triple-negative breast cancer, where different combinations of several cytotoxic drugs are usually chosen empirically by trial and error, the described device could provide a phenotypic readout of in vivo drug efficacy, which could be used to prioritize the given drugs for treatment.

## 4. Applications in Cardiovascular Diseases

Despite the steady developments in invasive cardiovascular interventions and pharmacological therapies, cardiovascular diseases (CVD) remain responsible for the majority of deaths worldwide. The term CVD covers a number of disorders of the circulatory system including atherosclerosis, thrombosis, and their clinical manifestations such as acute coronary syndrome, stroke, peripheral arterial disease, and venous thrombosis. On-demand drug delivery represents an important step toward the development of disease-targeted personalized therapies. Stimuli-responsive drug delivery systems offer the advantages of the disease site-specific treatment localization and a controlled drug release in the affected region, thus reducing off-target effects. Although the stimuli are commonly classified in three categories, namely physical, chemical, and biological, it is necessary to distinguish between the external stimuli (such as light, ultrasound, magnetic or electrical field) and intrinsic stimuli (usually chemical and biological, related to ionic strength, pH, or enzymatic reactions, but also biomechanical forces). The latter stimuli, being independent of an external trigger and having a higher sensitivity toward pathologic processes, are of particular interest in the biomedical field. Thus far, several stimuli-responsive drug delivery approaches to CVD have been reported including pH-, redox-, hypoxia-, and enzyme-responsive particles, polymers, or hydrogels. This review highlights some of the recently reported “smart” biosensing systems with therapeutic potential in CVD.

### 4.1. PH-Dependent Drug Delivery

The pH of the tissues is an important chemical parameter, changes of which are often related to the disease process. Consequently, this parameter can be exploited for phase-transition in polymer-based drug delivery systems in specific microenvironments or tissues affected by pathological processes. In line with this, DNA-based nanotubes were utilized as a drug carrier system for the pH-dependent delivery of dexamethasone [99]. The nanotubes loaded with glucocorticoid-conjugated oligonucleotides were rapidly internalized by mouse macrophages in vitro, and thanks to the presence of the pH-sensitive i-motif sequence, released dexamethasone in an acidic environment of the endolysosomal compartment. Compared with free dexamethasone, DNA-dexamethasone nanotubes significantly reduced the TNF-α expression in the LPS-stimulated macrophages in vitro. In a mouse model of ischemia-reperfusion, the administration of DNA-dexamethasone nanotubes into the post-ischemic muscle tissue led to reduced leukocyte transmigration and decreased the expression of the endothelial adhesion molecules.

### 4.2. Hypoxia-Sensing Drug Delivery Systems

Hypoxia is a driving mechanism of cell death in the ischemic tissues. On-demand drug delivery in response to hypoxia thus represents a promising approach to the treatment of myocardial ischemia. An interesting example of hypoxia-sensing drug delivery inspired by mitochondria was recently reported [100]. The double-shell poly(lactic-co-glycolic acid) (PLGA) nanoparticles contained melatonin to scavenge reactive oxygen species (ROS) and prevent apoptosis by activating mitochondrial melatonin receptor I to inhibit cytochrome c release. As a biological oxygen-sensing mechanism, circular DNA was incorporated on the surface of the particles by electrical adsorption. Oxygen-responsive vascular endothelial growth factor (VEGF) expression was realized by binding hypoxia-inducible factor-1α (HIF-1α) with erythropoietin enhancers and was shown to respond to alternating hypoxia–normoxia conditions by up- and downregulating the reporter expression. In a mouse model of myocardial infarction, these particles reduced cell death due to ischemia, improving the structural and functional capacity of the infracted hearts.

### 4.3. Reactive Oxygen Species-Responsive Drug Delivery

Atherosclerosis and its clinical manifestations including myocardial infarction, ischemia/reperfusion, or stroke are associated with an excessive generation of ROS. ROS have been implicated in the ischemic organ injury by inducing cell damage and apoptosis, but the systemic administration of exogenous antioxidants has proven to be ineffective against oxidative stress-induced injury. In the search for improved strategies of antioxidant delivery and ROS-sensitive targeting, several approaches have been reported. In a study by Li et al. [101], ROS-responsive nanoparticles produced of poly(ethylene glycol) (PEG) and poly(propylene sulfide) (PPS) were used for the encapsulation of a potent plant antioxidant, ginsenoside Rg3 (Rg3). Upon the exposure to ROS, Rg3-loaded PEG-b-PPS nanoparticles injected in the infarcted rat myocardium released Rg3, which improved the cardiac function by reducing the oxidative stress, inflammation, and fibrotic processes via the FoxO3a-dependent mechanism.

**Figure 6:**
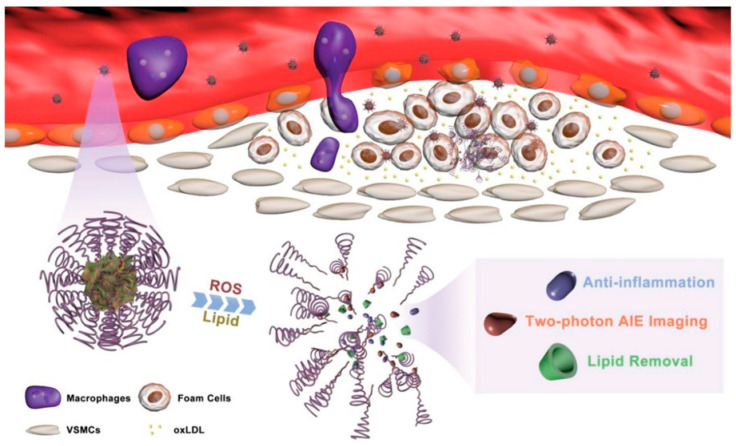
The ROS-responsive polymeric micelles release prednisolone in the lipid-rich environment of atherosclerotic plaque and allow for two-photon aggregation-induced emission imaging. Reproduced from Ma et al. [103], Open Access.

Another type of ROS-responsive theranostic nanoplatform was developed by Ma et al. [6] in order to detect and treat atherosclerotic plaques. Using a ROS-responsive bond, a fluorophore activated by two-photon aggregation-induced emission was linked to β-cyclodextrin. The system was loaded with prednisolone via supramolecular interaction and packed into nanosized micelles based on a ROS-sensitive copolymer poly(2-methylthioethanol methacrylate)-poly (2-methacryloyloxyethyl phosphorylcholine). The resulting micelles, termed TPCDP@PMM, have been shown to accumulate in the atherosclerotic plaques of ApoE^−/−^ mice and disrupt upon contact with ROS, leading to prednisolone release and atherosclerosis inhibition (Figure 6).

ROS-sensing drug delivery systems have also been developed for the treatment of stroke, which is responsible for about 10% of deaths worldwide and is a major cause of long-term disability. To achieve the specific targeting of a neuroprotective agent, NR2B9C, to the ischemic site, and improve the controllability of drug release, Lv et al. [102] produced a ROS-responsive nanocarrier. NR2B9C was loaded into dextran cores modified with a ROS-sensitive boronic ester to enable release triggered by high intracellular ROS in ischemic neurons.

Targeting of the nanosystem was realized by inserting a stroke homing peptide (CLEVSRKNC) into the shell composed of the erythrocyte membrane [102]. In vitro, the nanocarriers underwent a rapid hydrolysis in the presence of H_2_O_2_ and had strong protective effects against glutamate-induced cytotoxicity in the rat adrenal pheochromocytoma cell line PC-12. In a rat model of middle cerebral artery occlusion, ROS-sensing nanoparticles improved the active targeting of NR2B9C to the ischemic area and reduced ischemic brain damage.

The ROS-responsive polymeric micelles based on PEG-poly(tyrosine-ethyl oxalyl) (PEG-Ptyr-EO) that respond to the oxidative microenvironment of atherosclerotic plaques were also described [104]. The hyaluronic acid (HA) coating was designed to target CD44-positive inflammatory macrophages. In the presence of ROS, PEG-Ptyr-EO released simvastatin loaded into the particles to reduce the activation of plaque macrophages and additionally contributed to ROS consumption, thus diminishing the oxidative stress. Intravenous administration of ROS-responsive simvastatin-loaded micelles in a mouse model was shown to reduce the plaque cholesterol content and the burden of atherosclerosis.

### 4.4. Enzyme-Responsive Drug Delivery

Apart from oxidative stress, the microenvironment of atherosclerotic lesions and myocardial infarct tissue is characterized by an increased protease activity. This feature can be utilized to specifically deliver drugs to the regions with increased enzymatic activity if a cleavable, enzyme-responsive linker is introduced to the system. Wang et al. [105] reported a siRNA delivery system that responded to local upregulation in proteolytic activity after myocardial infarction. An injectable shear-thinning and self-healing hydrogel was composed of HA modified with hydrazides or aldehydes and combined with peptide crosslinkers that degrade in response to protease activity. HA was further modified with β-cyclodextrin to sequester cholesterol-modified siRNA against matrix metalloproteinase 2 (MMP2), which was implicated in adverse remodeling after myocardial infarction. In response to protease activity, the hydrogel eroded, releasing siRNA, which effectively silenced MMP2 expression in the cardiac fibroblasts in vitro. Compared to the hydrogels with the control siRNAs, the protease-sensing siMMP2 hydrogel led to significantly increased ejection fraction, stroke volume, and cardiac output in a rat model of myocardial infarction (MI). In parallel, it provided an improved mechanical support to the infarcted heart through reduced hydrogel erosion upon the silencing of MMP2, improving the myocardial thickness in the infarct at 4 weeks post-ischemic injury.

An interesting example of combining two distinct biological stimuli for the control of the drug delivery to atherosclerotic lesions was reported by Peters et al. [106]. The authors utilized peptide amphiphiles (PAs) to develop nanocarriers that sensed the increased levels of MMP2, MMP9, and ROS. To this end, the apolipoprotein A1-mimetic peptide was bound to PAs by peptide linkers cleaved by MMPs or ROS. The efficacy of the nanocarriers was tested in vitro on macrophages challenged with interferon gamma or lipopolysaccharide, showing the release of the ApoA1-Ac2–26 peptide and reduced macrophage activation.

A dual drug/siRNA delivery system responding to hyaluronidase type II (Hyal-2) was also reported [107]. The designed PLGA nanocarriers encapsulated atorvastatin to control lipid trafficking and reduce inflammation and siRNA against lectin-like oxidized low-density lipoprotein receptor-1 (LOX-1). The particle cores were clad in three external layers: an innermost lipid bilayer, an intermediate apolipoprotein A1 layer for macrophage targeting, and an outer layer of high molecular weight HA (200 kDa). The outer layer allowed CD44-dependent targeting, and upon Hyal-2 cleavage, led to exposure of the intermediate ApoA1 layer for enhanced entry into the macrophages. The efficacy of this dual-therapy nanosystem was demonstrated in atherosclerotic mice upon 12-week biweekly administration, showing a significant decrease in plaque size as well as reduced lipid and macrophage accumulation.

### 4.5. Shear-Responsive Systems

Although biomechanical forces play a major role in many pathophysiological processes, the strategies of targeting local alterations in physical forces associated with specific disease conditions have been poorly exploited [108]. In particular, in advanced atherothrombotic disease, characterized by stenotic atherosclerotic lesions that result in local increases in shear stress, mechanosensing drug delivery systems offer interesting possibilities for precise and localized therapies. Drug delivery to thrombus-occluded arteries by employing a mechanosensitive system was first described by Korin et al. [109]. The authors designed micro-aggregates of PLGA nanoparticles coated with thrombolytic drug tissue-plasminogen activator (tPA). These micro-aggregates remained stable under physiologic flow conditions (shear stress values up to 70 dyn/cm^2^), but experienced rapid break up followed by local drug release upon the exposure to abnormally high-shear stress in the regions of vascular occlusion. Compared with the free drug, the shear-activated tPA-coated nanoparticles rapidly dissolved arterial clots in mouse mesenteric arteries, with complete clearance of occluding thrombi within 5 min of administration. The doses of shear-activated tPA-nanoparticles required for clot dissolution were about 100-times lower than the doses required for achieving comparable effects with the free drug [109].

The mechanically-sensitive simvastatin release in the stenotic region was more recently described by Yao et al. [110]. To target inflammatory macrophages via the CD44 receptor, hydrogel micelles based on high-molecular weight HA modified with glycidyl methacrylate were developed. A cross-linkable block copolymer was used as a cross-linking agent and served in parallel as the drug coating material. The resulting HA-CBC micelles effectively targeted activated macrophages in vitro and underwent deformation and resizing under stenotic conditions (75% stenosis), leading to a drug release enhanced by about 50% compared to the non-stenotic conditions. The precise targeting of the fluorescently-labeled HA-CBC to macrophages was shown in ApoE^−/−^ mice and the effective release of simvastatin from HA-CBC micelles in the plaque region was confirmed in atherosclerotic rabbits [110].

The mechano-sensing strategy of drug delivery utilizing a universal hemodynamic phenomenon is expected to be applicable for all occlusive vascular conditions including the treatment of stenotic atherosclerotic plaques, pulmonary emboli, and ischemic stroke.

### 4.6. Advanced BioMEMs

Vascular stents improved the interventional treatment of atherosclerosis by achieving the immediate restoration of blood flow in obstructed arteries and providing mechanical support to the disease-affected vessels. However, stent implantation leads to disruption of the endothelial monolayer on the plaque surface, which may initiate acute thrombotic events. Furthermore, vascular wall injury resulting from stent implantation induces an excessive smooth muscle cell (SMC) proliferation, leading to restenosis.

To address the need of biological process monitoring, condition sensing, and controlled drug release, Son et al. reported a bioresorbable electronic stent (BES) containing a suite of sensors and actuators [111]. The stent struts were fabricated of bioresorbable/bioinert materials (ZM21 alloy, Mg 97%, Zn 2%, Mn 1%) and their surface was equipped with flow/temperature sensors as well as an embedded memory module for pattern analysis and diagnosis, incorporated in the outer polylactide (PLA) layer. Additionally, the system was equipped with ROS-scavenging ceria nanoparticles and rapamycin-loaded gold nanorod core/mesoporous silica shell nanoparticles that were activated by a local hyperthermia or an external optical stimulus (NIR laser). The hyperthermia was shown to control the localized rapamycin delivery for the suppression of SMC proliferation and enhance thermal therapy. The implantation of BES in a canine artery showed the inhibition of inflammatory responses and reduced macrophage infiltration. This innovative system combining mechanical, electronic, sensing, and therapeutic functionalities offers promising opportunities for the future stent designs.

## 5. Applications in Regenerative Medicine

Stimuli-responsive polymers have been employed in a variety of controlled or targeted drug delivery systems serving tissue regeneration. These “smart” polymers can respond to stimuli by network degradation or structure alteration, leading to the release of the drug payload [112,113]. However, similar to cardiovascular drug delivery, few systems to date have taken advantage of biomechanical stimuli from the microenvironment to initiate drug release. This mechanism is of particular importance in the regeneration of dynamic, actively moving tissues such as muscles or joints. To address this need, mechanically-activated PLGA microcapsules with different thresholds for mechano-activation, fine-tuned by changing the physical dimensions and composition, were reported by Mohanraj et al. [114]. The microcapsules were loaded with transforming growth factor-β3 (TGF-β3) and tested in an engineered cartilage model, demonstrating the release of the regeneration-supporting growth factor upon mechanical stimulus and an enhanced chondrogenesis in mesenchymal stem cells.

Arthritic disorders, affecting a growing number of patients, are characterized by low pH and the overexpression of MMPs. These biomarkers can therefore serve as stimuli for controlled drug delivery to osteoarthritis or inflammatory arthritis. As an example of this approach, nanomicelles containing an anti-inflammatory drug psoralidin were produced from biocompatible poly (2-ethyl-2-oxazoline)-poly (ε-caprolactone) (PeOx-PCL) [115]. The micelles were labeled with cartilage-targeting Coll-II α1 chain-binding peptide (WRYGRL) and conjugated with a specific peptide linker that was cleaved by MMP-13. Additionally, a fluorescent dye Cy5.5 and a quencher were coupled on the polymer micelles to obtain a theranostic platform. Upon the intra-articular injection in a mouse model of osteoarthritis induced by papain injection, MMP-13, which is upregulated in osteoarthritis, cleaved its substrate on the nano-micelles, leading to a fluorescent signal that reported active disease and confirmed the site-specific retention of particles. In parallel, the low pH of the osteoarthritis-affected joints resulted in gradual drug release upon the disassembly of micelles, preventing cartilage erosion and alleviating the severity of disease.

**Figure 7:**
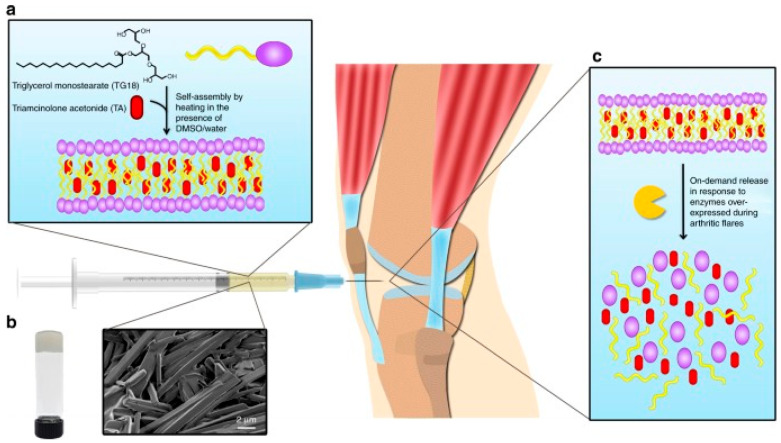
The biosensing triglycerol monostearate (TG-18) hydrogel releases the encapsulated drug (triamcinolone acetonide (TA) in response to enzymes upregulated during arthritis flares. (**a**). self-assembly of TG-18 to form hydrogel and encapsulation of TA; (**b**). scanning electron microscopy of TA-loaded TG-18 hydrogels; (**c**). disassembly of TG-18 hydrogel in response to flare-associated enzymes. The injectable hydrogel can be administered to the inflamed joints by intra-articular injection. Reproduced from Joshi et al. [116] Open Access.

Systemic pharmacologic therapies of inflammatory arthritis suffer from poor efficacy, as the amount of drug reaching the affected joints is low. However, the local delivery of therapeutics is also hampered due to their short half-lives in the articular tissue [116]. The development of a stimuli-sensitive drug delivery system that would respond to phases of high disease activity was described by Joshi et al. A triglycerol monostearate hydrogel forming self-assembled fibrous structures with interdigitated bilayers was loaded with triamcinolone acetonide (TA) and exposed to MMPs (MMP-2, MMP3, or MMP-9) or synovial fluid obtained from patients with rheumatoid arthritis. Under these conditions, the disassembly of hydrogel led to the release of the drug in an enzyme concentration-dependent manner. As a proof of concept, a hydrogel loaded with a fluorescent dye was injected in a mouse K/BxN serum-transfer model of arthritis, which induces arthritis flare in the forepaws and feet, showing a disease flare-dependent enzymatic digestion reflected in the loss of fluorescence. Compared to the free drug, a single dose of the TA-loaded hydrogel significantly suppressed the activity of arthritis in the injected paw (Figure 7).

Thermoresponsive polymers have been utilized in multiple applications related to tissue engineering and regenerative medicine including cell sheet technology [117], bioprinting polymers [118], and drug delivery. As an example of the latter, a delivery platform based on porous glycidyl methacrylated dextran/gelatin microcapsules combined with pore-filling thermo-responsive poly (*N*-isopropylacrylamide) (PNIPAAm) was reported [119]. The microcapsules were loaded with stromal cell-derived factor (SDF)-1α, an important chemokine that activates multiple signaling pathways involved in stem cell adhesion, migration, and proliferation, particularly in the context of bone formation and osteogenic differentiation. In response to temperature changes in vitro, swelling and shrinking of the PNIPAAm gates resulted in drug release from the microcapsules. Compared with non-thermoresponsive microcapsules, PNIPAAm capsules implanted subcutaneously in mice showed a sustained long-term release of SFD-1, which is of importance for the effective regeneration of the affected tissues.

The light-activated modulation of cell function is a novel approach to tissue regeneration. Thus far, the possibilities of photoreceptor activation by ultraviolet or visible light have been limited due to the poor penetration of thicker tissues. This limitation can be overcome by a near-infrared light (NIR)-activable nanosystem modulating native receptor tyrosine kinase (RTK), as recently reported by Wang et al. [120]. The developed therapeutic platform was composed of gold nanorods (AuNRs) conjugated via thiol-gold chemistry with an inactivated DNA agonist, which is released and activated upon NIR treatment that induces the localized surface plasmon resonance-based photothermal effect on AuNRs. After the activation, the DNA agonist binds to the cell membrane RTK, resulting in homo- or hetero-dimerization of RTKs and subsequent tyrosine autophosphorylation. This initiates the downstream signaling cascades that control cell migration, polarization, and cytoskeleton reorganization. In a proof-of-concept study in vivo, the nanorods were shown to mediate RTK signaling and induce skeletal muscle satellite cell migration and myogenesis. This type of stimuli-responsive receptor-mediated control of cell function can prove beneficial in the regeneration of skeletal muscle and other tissues.

**Figure 8:**
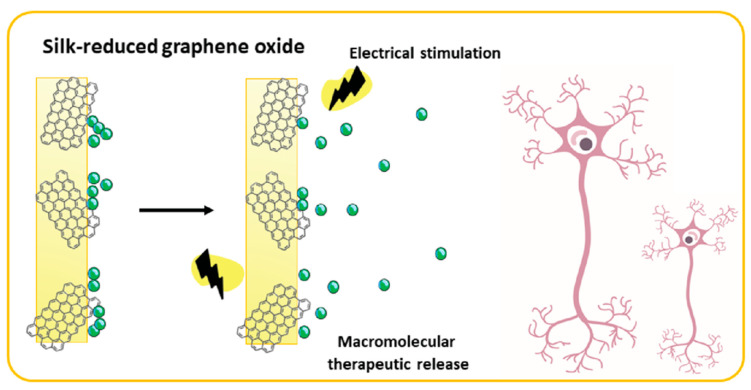
The composite silk fibroin and graphene oxide films have electroconductive properties and allow for loading and the controlled release of nerve growth factor-β upon the application of an electrical stimulus. Reproduced from Magaz et al. [121], Open Access.

Neural tissue regeneration represents the biggest challenge in the field of regenerative medicine. Coupling drugs with responsive materials for the precise control of therapeutic retention and release is, therefore, a promising approach that could overcome the limitations of current therapies. Composite films, incorporating a natural biopolymer Bombyx mori silk fibroin and electroconductive component that reduced the graphene oxide, were recently developed for electrostatic loading with a macromolecular therapeutic, nerve growth factor-β (bNGF) [121]. Upon application of an electrical stimulus, the growth factor-loaded films released the bNGF over a 10-day period in a controllable fashion. This type of biologically responsive scaffold holds a great promise for neural tissue regeneration, although the in vitro and in vivo data are yet to confirm its safety and efficacy (Figure 8).

Taken together, on-demand drug delivery is often the key to therapeutic success, but is very limited without the ability of nanocarriers to sense and respond to the diseased microenvironment. Achieving a precise control over therapeutic agents, in terms of targeting, retention, and stimuli-sensitive release over long-term periods is of particular importance in order to regulate the biological repair process and promote tissue regeneration.

## 6. Conclusions

Biosensor-integrated drug delivery systems have been a multidisciplinary technological advancement that allows for both the monitoring and delivery of the drug with precision. Several technologies were described in this review for chronic diseases (e.g., diabetes, cancer, cardiovascular diseases) and regenerative medicine, which promise personalized treatment favoring clinical success. The gold standard for the treatment of diabetes would be wearable biosensors with improved specificity and sensitivity, and of a low detection limit for the precision delivery, aside from overcoming the challenge of the oral delivery of insulin. Regarding cancer treatment, the use of smart nanomotors with a biosensing and delivery capacity at the cellular level would revolutionize chemotherapy in the short-term. Wearable devices for pre-programmed intravenous drug delivery would reduce adverse side effects as the systemic drug exposure would be lower. Although the cost is still relatively high, significant benefits have been reported with the use of continuous monitoring, together with the controlled delivery of the precise drug dose to the target, thus a higher compliance to the treatment, also attributed to the site-specific drug delivery. The challenges encountered with the biosensors are their small size, sensitivity, and low limit of detection. Overcoming these limitations, this integrated system opens the possibility in the near future of using several smart biomaterials of organic and inorganic origin, together with drugs of different physical and chemical properties to improve the diagnosis, treatment, and management of diseases.

## Data Availability

Not applicable.
